# High Mobility Group Box 1 Expression in Oral Inflammation and Regeneration

**DOI:** 10.3389/fimmu.2020.01461

**Published:** 2020-07-14

**Authors:** Keisuke Yamashiro, Hidetaka Ideguchi, Hiroaki Aoyagi, Chiaki Yoshihara-Hirata, Anna Hirai, Risa Suzuki-Kyoshima, Yao Zhang, Hidenori Wake, Masahiro Nishibori, Tadashi Yamamoto, Shogo Takashiba

**Affiliations:** ^1^Department of Periodontics and Endodontics, Okayama University Hospital, Okayama, Japan; ^2^Department of Pathophysiology-Periodontal Science, Okayama University Graduate School of Medicine, Dentistry and Pharmaceutical Sciences, Okayama, Japan; ^3^Department of Pharmacology, Okayama University Graduate School of Medicine, Dentistry and Pharmaceutical Science, Okayama, Japan

**Keywords:** high mobility group box 1, inflammation, periodontal regeneration, periodontitis, osseointegration, tooth movement, wound healing

## Abstract

High mobility group box 1 (HMGB1) is a non-histone DNA-binding protein of about 30 kDa. It is released from a variety of cells into the extracellular milieu in response to inflammatory stimuli and acts on specific cell-surface receptors, such as receptors for advanced glycation end-products (RAGE), Toll-like receptor (TLR)2, TLR4, with or without forming a complex with other molecules. HMGB1 mediates various mechanisms such as inflammation, cell migration, proliferation, and differentiation. On the other hand, HMGB1 enhances chemotaxis acting through the C-X-C motif chemokine ligand (CXCL)12/C-X-C chemokine receptor (CXCR)4 axis and is involved in regeneration. In the oral cavity, high levels of HMGB1 have been detected in the gingival tissue from periodontitis and peri-implantitis patients, and it has been shown that secreted HMGB1 induces pro-inflammatory cytokine expression, such as interleukin (IL)-1β, IL-6, and tumor necrosis factor (TNF)-α, which prolong inflammation. In contrast, wound healing after tooth extraction or titanium dental implant osseointegration requires an initial acute inflammation, which is regulated by secreted HMGB1. This indicates that secreted HMGB1 regulates angiogenesis and bone remodeling by osteoclast and osteoblast activation and promotes bone healing in oral tissue repair. Therefore, HMGB1 can prolong inflammation in the periodontal tissue and, conversely, can regenerate or repair damaged tissues in the oral cavity. In this review, we highlight the role of HMGB1 in the oral cavity by comparing its function and regulation with its function in other diseases. We also discuss the necessity for further studies in this field to provide more specific scientific evidence for dentistry.

## Introduction

An inflammatory or immune response in oral tissues occurs when biological reactions such as microbial infection, physical trauma, neoplastic processes, and autoimmune conditions occur in the oral environment. The oral cavity contains complex microbial flora, and the immune system promotes pro-inflammatory cytokine production (1). Sterile injuries, such as tooth extraction and orthodontic tooth movement also cause an immune reaction and subsequent tissue repair. Immediately after a dental extraction is performed, blood platelets are recruited into the collagen connective tissue, a clot starts to form, and growth factors and angiogenesis mediators are being produced (2). In the context of orthodontic tooth movement, an initial inflammatory reaction is generated at the compression sites caused by constriction of the periodontal ligament. The periodontal ligament releases pro-inflammatory cytokines and promotes tissue resorption (3). It has been mentioned that chronic inflammation and oxidative stress promote carcinogenesis (4). In the oral cavity, it has been suggested that both cyclooxygenase (COX) 2 and chronic inflammation are involved in the initiation of carcinogenesis process of oral squamous cell carcinoma (5). Becht's disease, a well-known autoimmune condition, exhibits symptoms such as aphthous ulcers in the oral cavity, and it is suggested that there is a relationship between this disease and periodontitis, which is a major chronic oral inflammatory pathology (6). It is thought that the inflammatory and immune response are closely related to both the progress of the diseases and tissue repair.

HMGB1 is a nuclear protein that regulates transcription and is one of the damage-associated molecular patterns (DAMPs), which act as major mediators in immune reactions. HMGB1 has several isoforms, which have distinct biological implications. These isoforms are: “fully reduced HMGB1,” “disulfide HMGB1,” and “sulfonyl HMGB1” named after the different redox reactions that occur in the three cysteines at positions 23, 45, and 106 of HMGB1 (7). Necrotic cell death or cell stress promotes fully reduced HMGB1, which forms a heterocomplex with CXCL12. The heterocomplex binds to the CXCR4 receptor with increased affinity and enhances chemotaxis (8). Fully reduced HMGB1 can be oxidized to disulfide HMGB1, which forms a disulfide bond between C23 and C45, and exerts a pro-inflammatory effect by promoting cytokines production via the TLR4/myeloid differentiation factor 2 (MD-2) complex. Fully oxidized HMGB1 and sulfonyl HMGB1 are thought to be inert (9, 10). The difference in the isoforms is thought to be one of the reasons why HMGB1 is involved in two opposing functions: progression of inflammation and tissue repair.

Initially, HMGB1 was shown to cause a danger signal in acute inflammatory diseases such as sepsis. Wang et al. (11) reported that HMGB1 was liberated from cells stimulated with cytokines and that HMGB1 plays an important role in mediating experimental sepsis. Yamamoto et al. (12) reported that lipopolysaccharide (LPS) increased pro-inflammatory cytokine secretion from peritoneal macrophages and initiated intracellular signaling to activate nuclear factor kappa-light-chain-enhancer of activated B cells (NF-κB) by binding RAGE and TLR2. In the report, they mentioned that LPS-mediated RAGE signaling accelerates acute inflammation and vascular dysregulation, leading to tissue damage, which then mediates HMGB1 release in the late phase, resulting in a pernicious cycle of RAGE-dependent lethality in septic shock. Yang et al. (9) reported that disulfide HMGB1 binds MD-2/TLR4, and MD-2 antagonists inhibited hepatic ischemia/reperfusion injury, chemical toxicity, and sepsis in mice. HMGB1 is not only involved in acute inflammation but also in chronic inflammation. Gasiorowski et al. (13) reported that RAGE activation should be perceived as a primary mechanism that determines self-perpetuated chronic inflammation in Alzheimer's disease, and the crosstalk; RAGE cooperation with TLRs amplifies inflammatory signaling via extracellular signal-regulated kinase (ERK)1/2, mitogen-activated protein kinase (MAPK), p38, c-Jun N-terminal kinase (JNK), and NF-κB signaling. In a recent report, it was shown that the HMGB1/CXCL12 heterocomplex can be maintained in rheumatoid arthritis (RA) by the activity of the prostaglandin E2 (PGE2)/COX2 pathway, the Janus kinase/signal transducer and activator of transcription (JAK/STAT) pathway, and the thioredoxin system, all of which are associated with the activation of the disease (14). In addition, in juvenile idiopathic arthritis patients (JIA), the presence of three functional HMGB1 redox isoforms confirms the complexity of their pathogenic role during chronic inflammation (15).

Until now, many researchers have focused on HMGB1 as an inflammatory mediator that prolongs various inflammatory diseases, and because of this research has focused on inhibiting the function of HMGB1 to treat such diseases (16, 17). However, in recent reports, another aspect of HMGB1, which is related to its role in tissue healing and regeneration, is being highlighted (18, 19). Originally, inflammation is believed to be not only a chronic and degenerative disease, but also part of the physiological process that initiates tissue repair and regeneration. Infection or injury of epithelium leads to the generation of DAMPs and pathogen-associated molecular patterns (PAMPs), which then activate immune cells for regeneration by stimulating cell proliferation and differentiation (20). Vénéreau et al. (18) created the mutant HMGB1 (3S HMGB1), in which the cysteines are replaced with serines, which are resistant to oxidation, and behave as reduced HMGB1. Tirone et al. (21) reported that 3S HMGB1 orchestrates muscle and liver regeneration via CXCR4. A recent study also reported that fully reduced HMGB1 forms a heterocomplex with CXCL12, which binds to CXCR4 and then accelerates skeletal, hematopoietic, and muscle regeneration (22).

In addition, an explanation of the HMGB1 function is that HMGB1-C1q complexes regulate macrophage polarization by inducing the differentiation to anti-inflammatory M2-like macrophages (23). The activation of complements is strongly involved in immune cell migration. There are three pathways of complement activation: the classical pathway, the Mannan-binding lectin pathway, and the alternative pathway, all of which promote immune cell migration by producing the cleaved complement component 3 (C3a) and cleaved complement component 5 (C5a) (24). On the other hand, the C1 complex (C1q), which normally triggers the classical pathway, is thought to regulate both inflammation and regeneration by the coexistence of DAMPs. Liu et al. (25) reported that the HMGB1-C1q complex induces production of proresolving mediators such as resolvin (Rv)D1 and RvD2. The resolution of inflammation and macrophage polarization may result in tissue regeneration.

The oral cavity has a complex environment having a variety of different tissues such as epithelium, connective tissue, and hard tissue such as teeth and bone, along with various bacterial species. Thus, in this complex environment HMGB1 may play its dual role in prolonged inflammation and tissue regeneration. However, we still do not know the detailed mechanism by which the isoform of HMGB1, or how the HMGB1 forming complex is involved in oral inflammation and regeneration in the oral cavity. In this review, we specifically focus on the role of HMGB1 in oral inflammation and regeneration. We introduce past reports and suggest future directions.

## HMGB1 in Oral Inflammatory Conditions

### Periodontal Inflammation

Periodontal diseases are dysbiotic conditions in the gingival margin, which are characterized by an imbalance between subgingival microbial communities and the host immune response (26, 27). Clinical studies have demonstrated that the levels of TLR2 and TLR4 in periodontitis patients were significantly higher than those in control groups (28, 29). Li et al. (29) also demonstrated the presence of TLR4, CD14, and MD-2 expression in both cultured human gingival keratinocytes and fibroblasts. *Porphyromonas gingivalis* (*P. gingivalis*) is considered a keystone pathogen for periodontitis (30). *P. gingivalis* fimbriae can activate the TLR2 and TLR4 pathways, leading to excessive production of pro-inflammatory cytokines and chemokines in monocytic cells (31). PAMPs are conservative molecules associated with groups of pathogens or their products, and are involved in the development of periodontitis. LPS of *P. gingivalis* is an effective ligand for TLR4, and Li et al. (32) discovered that the ability of human periodontal ligament stem cells (hPDLSCs) to differentiate into osteoblasts was impaired by LPS, through a TLR4-mediated NF-κB pathway. Lipoprotein derived from *P. gingivalis* can serve as a ligand for TLR2 and activate the NF-κB pathway (33). Okugawa et al. (34) reported that soluble peptidoglycans (PGNs) of *P. gingivalis* and *Aggregatibacter actinomycetemcomitans* (*A. actinomycetemcomitans)* induced IL-8 production in cultured oral epithelial cells. TLRs on gingival tissues such as gingival epithelium, gingival fibroblasts, periodontal ligaments, and immune cells recognize PAMPs, and the TLR signals induce significant amounts of inflammatory mediators. An exaggerated reaction response by the immune response promotes the production of receptor activator of nuclear factor kappa-B ligand (RANKL), activates osteoclasts, and then cause tissue destruction and bone resorption.

### The Cells in Periodontal Tissue Produce HMGB1

Not only PAMPs but also alarmins such as HMGB1 are considered as a significant factor during osteoclastogenic. Infection promotes HMGB1 secretion from periodontal tissue, and the secreted HMGB1 is involved in the lingering or aggravation of periodontitis. HMGB1 was detected at high levels in gingival crevicular fluid (GCF) in periodontitis patients (35, 36). There were significant positive correlations between the levels of HMGB1 in GCF and all periodontal parameters, including plaque index, bleeding index, probing depth, and clinical attachment level. The abundance of HMGB1 in GCF in chronic periodontal patients suggests that human gingival epithelial cells secrete HMGB1 up on stimulation by bacterial infection (37). That study also confirmed that TNF-α promotes HMGB1 production *in vitro*, using rat gingival epithelial cells and Ca9-22 cells, which is an oral epithelial cell line. Ito et al. (38) reported that IL-1β promoted the secretion of HMGB1 in human gingival epithelial and fibroblast cells. They also confirmed that gene expression of RAGE was highly upregulated by IL-1β stimuli in cultured human gingival epithelial cells, and that HMGB1 and RAGE were highly expressed in gingival epithelial cells in patients with oral inflammation. Another study reported that HMGB1 was dislocated from the nucleus of the cells in the pocket epithelium, which faces the infected root surface, but it was mainly localized in the nucleus in the gingival epithelium of periodontitis patients (39). They also confirmed that butyric acid, which is a metabolite of periodontal pathogens and a virulence factor of *P. gingivalis*, induced HMGB1 production in Ca9-22 cells *in vitro*. Our previous report suggested that HMGB1 translocated from the nucleus into the cytoplasm in the gingival epithelium *in vivo*, in a periodontal mouse model with *P. gingivalis*-soaked ligatures (40). *In vitro* analysis using cultured progenitor human gingival epithelial cells (HGECs) and THP-1 cells, which is a macrophage-like cell line, showed that TNF-α induced HMGB1 production. Interestingly, the amount of HMGB1 production was lower in HGECs (<20 ng/mL) than in THP-1 cells (more than 60 ng/mL) (40). Gingival connective tissue located between the epithelium and the root surface contains gingival fibroblasts. It has been reported that cultured human gingival fibroblasts (HGF) produce HMGB1 upon stimulation by LPS of *A. actinomycetemcomitans, P. gingivalis*, and *Escherichia coli*, and upon apoptotic and necrotic initiation (41). The periodontal ligament, which is also a connective tissue lying between the alveolar bone and tooth root, contains periodontal ligament fibroblasts (PDLF). Nogueira et al. (42) reported that HMGB1 was produced in cultured human PDLF upon treatment LPS and IL-1β. They also confirmed the expression of HMGB1 in the periodontal ligament in an experimental periodontitis rat model (42). However, we still do not know which tissue is the main source of HMGB1 production in periodontium.

Secretion of HMGB1 around periodontal tissue is considered to promote pro-inflammatory cytokine production and prolong periodontitis. Kim et al. (43) reported that recombinant HMGB1 induced the expression of TNF-α, IL-1β, IL-6, IL-11, and IL-17 mRNA in immortalized human PDL cells (hPDLCs). They also showed that TLR4 and TLR2 expression was increased in hPDLCs exposed to HMGB1 and that neutralizing anti-TLR2 and anti-TLR4 antibodies specifically inhibited HMGB1-induced expression and secretion of osteoclastogenic cytokines and expression of RANKL (43). Parks et al. (44) reported that RAGE plays only a minor role in macrophage activation by HMGB1, whereas signaling through TLR 2 and TLR4 prompted the release of TNF-α, IL-1β, and IL-6 from cultured mouse neutrophils and macrophages. In addition to other inflammatory diseases, secreted HMGB1 is considered to promote inflammation; however, there is little evidence to have examined the difference in HMGB1 isoforms in this research area. One of the reasons for these limitations was the difficulty in using recombinant HMGB1 proteins because they easily form complexes with other molecules such as LPS and IL-1β.

Periapical lesions are osteolytic bone defects that are inflammatory immune defensive reactions that originate as a consequence of microbial and toxin invasion into the root canal. It has been established that a variety of cytokines and chemokines participate in the innate immune-inflammatory response and later in the adaptive immune response (45). These cytokines, including matrix metalloproteinases (MMPs), IL-1β, and IL-6 play a critical role in osteoclast formation and alveolar resorption (46). Liu et al. (47) reported the presence of HMGB1 and TLR4-positive cells around periapical lesions surrounding the apical foramen via osteoclast activation.

In addition to teeth, dental implants also suffer from bacterial infection. Peri-implantitis is a destructive inflammatory process caused by bacteria surrounding dental implants (48). Previously, it was reported that RAGE levels were elevated in patients susceptible to periodontitis compared to healthy patients, but TLR2 and TLR4 levels did not change before implant therapy. After implant therapy, RAGE and TLR4 levels were upregulated but TLR2 levels were downregulated (49). In addition, a higher concentration of HMGB1 has been found in the GCF from inflammatory gingival tissue in comparison to the healthy site around the dental implant (35, 50). The expression levels of other pro-inflammatory factors, such as IL-1β, Il-6, IL-8, and TNF-α, were also higher in the GCF from peri-implantitis sites than in the GCF from healthy sites. They reported that HMGB1 expression level in GCF is indicative of the progression of peri-implantitis and may be a useful diagnostic biomarker.

### HMGB1 Blockade Inhibits Periodontitis Progression

It was revealed that HMGB1 is involved in the aggravation of periodontitis by HMGB1 blockade analysis. Glycyrrhizin is the chief sweet-tasting constituent of *Glycyrrhiza glabra* root and is contained in various oral hygiene products such as toothpaste and mouth wash to exert an anti-inflammatory effect (51, 52). Mollica et al. (52) reported that glycyrrhizin binds to HMGB1 specifically and inhibits cytokine activity. In our previous report, the progression of periodontitis was inhibited in a mouse periodontitis model with glycyrrhizin (53). A recent study reported that glycyrrhizic acid suppressed inflammation and reduced the increased glucose levels induced by the combination of *Porphyromonas gulae* and ligature placement in mouse model of diabetes (54). In this study, glycyrrhizic acid also suppressed ligature/*P. gulae*-induced increases in HMGB1 and RAGE both at the mRNA and serum levels in the gingiva of diabetic mice. The anti-HMGB1 antibody is one of the most powerful HMGB1 inhibitors and has been used in many inflammatory disease models such as sepsis and brain infarction (11, 55). In our study, administration of anti-HMGB1 antibody in a murine periodontitis model inhibited myeloperoxidase (MPO) activity, neutrophil migration, and bone resorption in a dose-dependent manner. This result suggested that a faster resolution of periodontal inflammation can be achieved by blocking HMGB1. The antibody inhibited the expression of IL-1β and C-X-C motif chemokine ligand 1 (CXCL1) in cultured cells. The antibody also inhibited TNF-α-induced IL-1β production in HGECs and TNF-α-induced GM-CSF production in THP-1 cells *in vitro* (40). In early inflammation, gingival epithelial cells release various cytokines and chemokines, and HMGB1 is then translocated from the nucleus to the cytoplasm upon stimulation by TNF-α. The released HMGB1 induces the translocation in an autocrine-related manner; the released HMGB1 also induces GM-CSF secretion from gingival epithelial cells, resulting in the differentiation and activation of immune cells. As inflammation proceeds via the continuous secretion of HMGB1, macrophages release more cytokines, chemokines, and HMGB1. Moreover, the released IL-1β promotes osteoclastogenesis and bone resorption. Therefore, periodontal inflammation is initiated, exacerbated, and prolonged by the HMGB1 secretion cycle. The present study demonstrated that anti-HMGB1 antibody succeeded in preventing prolonged immunostimulation and bone-resorbing activity of osteoclasts by inhibiting the release of cytokines in periodontal tissue. However, HMGB1 blockade with anti-HMGB1 antibody partially inhibited periodontal progression, thus indicating that there might be another HMGB1-independent pathway.

Periodontitis has been associated with many other systemic diseases; for instance, there is a two-way relationship between diabetes and periodontitis (56). In 2012, a hypothesis was reported that secreted HMGB1 acting through RAGE, on monocytes, macrophages, and vascular endothelial cells, and might play an important role in the development of diabetes-associated periodontitis (57), and many reports regarding this relationship are currently being conducted. RAGE is one of the receptors for HMGB1, and its expression is higher in gingival tissue of patients with type 2 diabetes than in healthy patients (58). Blockade of HMGB1 by soluble RAGE (sRAGE) suppressed periodontitis-associated bone loss in diabetic mice (59), and serum levels of sRAGE and cleaved RAGE were significantly lower in periodontitis patients (60). However, soluble RAGE to neutralize RAGE receptors does not specifically block HMGB1, as there are multiple other RAGE ligands, such as AGEs, S100As, and lysophosphatidic acid (LPA), which may bind to this receptor as well as to HMGB1 (61, 62). Metformin, the first-line medication for the treatment of type 2 diabetes, is also considered an HMGB1 inhibitor because it directly binds HMGB1 and inhibits the pro-inflammatory activity (63). Metformin and metformin hydrochloride-loaded poly lactic-co-glycolic acid nanoparticles decreased the inflammatory response and bone loss in a rat periodontitis model (64, 65). These findings indicate that metformin does not only have a hypoglycemic action but also has an anti-inflammatory effect to block HMGB1, and it might be effective in diabetes-associated periodontitis. In summary, HMGB1, and RAGE are involved in the two-way relationship between diabetes and periodontitis, and metformin has the potential to resolve them.

## Oral Regeneration Associated With HMGB1

### Wound Healing Around the Gingival Tissue and After Tooth Extraction

Tancharoen et al. (66) reported that HMGB1 promotes intraoral palatal wound healing through RAGE. *In vivo* analysis showed that the wound closure of palatal gingival tissue was attenuated in heterozygous HMGB1 (*Hmgb1*^+/−^) mice compared to wild type (WT) mice. In the *Hmgb1*^+/−^ mice, the number of proliferating cell nuclear antigen (PCNA), NF-κB p50, and vascular endothelial growth factor (VEGF) were lower than those in WT mice. *In vitro* analysis using cultured HGF revealed that keratinocyte proliferation and migration during re-epithelialization was delayed in RAGE knockdown cells compared to that of the control, as determined by the wound scratch assay and the gene expression level of PCNA. Bone healing after tooth extraction is representative of intramembranous ossification. Healing after tooth extraction requires an initial acute inflammation, which is regulated by secreted HMGB1. Anti-HMGB1 antibody inhibits MPO activity, IL-1β and VEGF-A expression, and migration of CD31-, CD68-, TRAP-, and osteocalcin-positive cells. This indicates that the secreted HMGB1 regulates angiogenesis and bone remodeling by osteoclast and osteoblast activation, thus promoting bone healing in the tooth extraction socket (67). This indicates that anti-HMGB1 antibody inhibits not only aggravation of inflammatory diseases such as periodontitis, but also inhibits initial acute inflammation, which is essential for tissue repair.

### Periodontal Regeneration

Periodontal regeneration is defined histologically as regeneration of the tooth's supporting tissues, including alveolar bone, periodontal ligament, and cementum on a previously diseased root surface (68). Particularly, PDLFs are thought to play an important role in periodontal wound healing and regeneration because they contain stem cells that can be used to regenerate periodontal tissues (69). HMGB1 protein induced PDLF proliferation and migration in *in vitro* studies. It also promoted osteogenic differentiation parameters such as alkaline phosphatase (ALP), osteopontin, osteocalcin, RUNX2, and bone morphogenetic protein (BMP) (70). However, the authors considered that HMGB1 might support the reestablishment of the structural and functional integrity of the periodontium, following periodontal trauma such as orthodontic tooth movement described later, and might not support periodontal regeneration. Indeed, there is no evidence that HMGB1 is involved in periodontal regeneration. For further study, it must be explored whether the different isoforms of HMGB1 are involved in periodontal regeneration.

### Orthodontic Tooth Movement

In addition to the *in vitro* analysis with PDLF (70), Wolf et al. (71) also indicated that HMGB1, initially produced in PDLFs by mechanical loading during orthodontic tooth movement, decreased gradually. Initial HMGB1 production enhances the activity of monocytes and macrophages by clearing cellular debris and activating RANKL to initiate bone remodeling (71). Cui et al. (72) also reported that mechanical stress during orthodontic tooth movement induces PDLFs to secrete HMGB1. Mechanical stress also induced pro-inflammatory cytokine expression, such as TNF-α and IL-6, from macrophages, which activates the innate immune response. HMGB1 and these pro-inflammatory cytokines are reduced in a time-dependent manner (72). HMGB1 is thought to react in an acute innate immune response, and the gradual reduction of HMGB1 in PDLF is necessary for the achievement of orthodontic tooth movement; however, more evidence is still needed.

### Dental Pulp Regeneration

Many studies regarding tissue regenerative procedures have found that dental pulp cells (DPCs) are one of the stem cell sources (73, 74). Zhang et al. (75) showed that in healthy dental pulps, HMGB1 remains in the nuclei (confirming its nuclear localization), but in inflamed pulps, the presence of HMGB1 in the cytoplasm of infiltrated inflammatory cells, fibroblasts, and endothelial cells increases. Moreover, HMGB1 mRNA levels in these cells have been demonstrated to increase, which means that the pulp infection also stimulates the synthesis of this molecule. Through *in vitro* studies, these authors have also demonstrated that elevated cytoplasmic presence of HMGB1 mRNA levels after *E. coli* LPS stimulation in cultured DPCs. It has also been demonstrated that high levels of cytokines in pulpitis such as IL-6, IL-1, and TNF-α are also released by HMGB1 secretion (76). Some studies have concluded that HMGB1 and its receptor, RAGE, are involved in stem/progenitor cell differentiation in order to repair damaged tissues (77, 78). In a study by Zhang et al. (75) it was demonstrated that HMGB1 promoted DPCs migration in a dose-dependent manner, and that HMGB1 also activated Rho signaling and cytoskeletal reorganization. Thus, the formation of new dentin could be established, confirming the findings from Qi et al. (79) who found that HMGB1 promotes odontoblast differentiation from DPCs. However, excessive quantities of this molecule may amplify inflammation and may cause tissue damage. Based on these findings, it can be concluded that HMGB1 plays crucial roles not only in dental pulp inflammation, but also in dentine regeneration, enhancing DPC recruitment into the pulp injury, stimulating their differentiation into odontoblasts, and new dentin formation for healing of damaged tissues. Furthermore, we recently reported that RvD2 induces active resolution of inflammation through pulp-like tissue regeneration after root canal infection (80). It is possible that the HMGB1-C1q complex induces the production of RvD2 for dental pulp regeneration, as suggested by Liu et al. (25).

### Ti Osseointegration

Interestingly, in the latest report, HMGB1 is involved not only in peri-implantitis but also in osseointegration. Osseointegration is defined as the direct structural and functional connection between the living bone and the surface of a load-bearing artificial dental implant. Biguetti et al. (81) reported that the released HMGB1 binding to RAGE contributes to titanium (Ti)-mediated osseointegration in dental implants. In this report, HMGB1 was detected at high levels at bone Ti implantation sites immediately after implantation, followed by a gradual decrease in later time points. Inhibition of HMGB1 with glycyrrhizic acid and RAGE antagonistic peptide decreased bone matrix formation, blood vessel formation, and migration of osteoblasts and osteoclasts around the Ti surface. The growth factors and mesenchymal stem cell markers were upregulated in the oral osteointegration model, but these were reduced in the HMGB1 inhibition models.

## Conclusion and Future Directions

**Figure 1:**
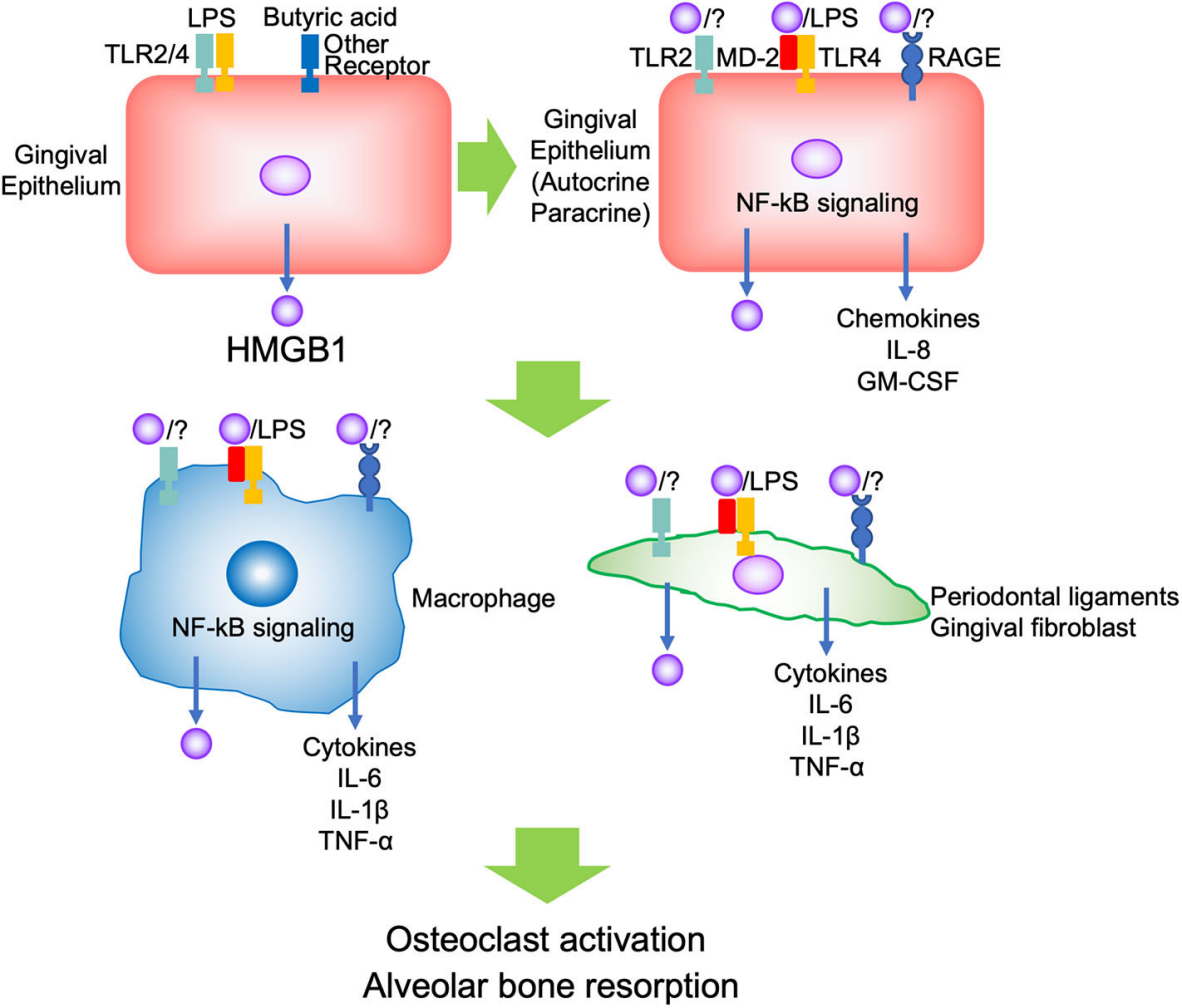
Scheme of the role of HMGB1 in periodontitis progression.

Evidence indicates that HMGB1 is associated with inflammation or immune response in both pathogenic and repair processes in the oral cavity. The reason may depend on the amount and duration of HMGB1, and the kind of stimulus, such as whether the conditions were sterile or infectious, not its dual role. In the context of periodontal inflammation, PAMPs such as LPS initially bind TLR2/4 and butyric acid binds to other receptors on the gingival epithelium, and promotes HMGB1 secretion. The secreted HMGB1 forms a complex with other molecules such as LPS, binds TLR2/4 and RAGE on the adjacent epithelium, thus promoting autocrine/paracrine signaling. The inflamed epithelium produced pro-inflammatory cytokines and chemokines such as IL-8 and GM-CSF to migrate and differentiate immune cells. Migrated immune cells, such as neutrophils and macrophages, are activated by secreted HMGB1, and produce pro-inflammatory cytokines such as IL-1β, IL-6, TNF-α, and HMGB1 via TLR2/4 and RAGE. These cytokines induce further HMGB1 production from other tissues, such as gingival fibroblasts and periodontal ligaments. Then, the aggravated inflammation promotes osteoclastogenesis and causes alveolar bone resorption (Figure 1). There was no evidence regarding which isoform of HMGB1 was mostly involved in the progression of periodontal disease. In JIA, which is characterized by chronic inflammation and periodontitis, it was indicated that the presence of various functional HMGB1 redox isoforms confirms the complexity of their pathogenic role during chronic inflammation (15). Thus, subsequent studies should focus on improving the understanding of the biological effects of different isoforms of HMGB1 and different receptors in periodontitis.

**Figure 2:**
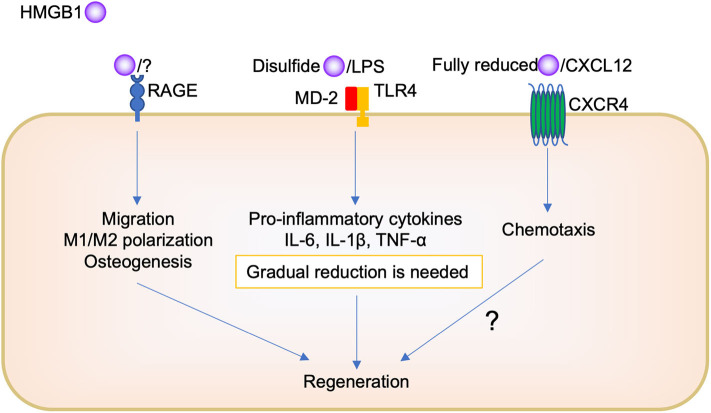
Scheme of the role of HMGB1 in oral regeneration.

On the other hand, regarding tissue repair, it is believed that because of the complexity of HMGB1, its different pathways depending on the redox forms, and its complex formation with other molecules, it is difficult to know how it orchestrates its biological function (Figure 2). The administration of HMGB1 antibody inhibited chemotaxis, such as neutrophil and macrophage migration, during socket repair. Tirone et al. (21) reported that fully reduced HMGB1 induced muscle and liver regeneration via CXCR4, whereas “disulfide HMGB1” and its receptors TLR/MD-2 and RAGE are not involved. However, we believe that HMGB1/RAGE signaling is also important in oral tissue repair. Keratinocyte proliferation and migration during oral palate healing are regulated by HMGB1/RAGE signaling (66). Biguettiet al. (81) reported that HMGB1/RAGE signaling is involved in stem cell migration, macrophage M1/M2 polarization, and osteogenesis during Ti-implant osseointegration. In addition, further studies are needed to examine whether fully reduced HMGB1, CXCL12, and CXCR4 participate in the biological process.

Furthermore, there is need to produce a certain amount of HMGB1; disulfides HMGB1 binding TLR2 or TL4, to produce pro-inflammatory cytokines for initial inflammation during tissue repair. The presence of oral bacteria may be important to determine whether HMGB1 plays a role in inflammation or regeneration in the oral cavity. Pathogen removal by physical approach or immune cell activity such as phagocytosis decreases HMGB1 secretion following pro-inflammatory cytokine reduction, M1/M2 polarization change, and then promotes tissue repair. However, the remaining pathogen induces further HMGB1 secretion, continued pro-inflammatory cytokine production, and impaired healing or chronic inflammation. To understand the detailed mechanism of this complexity of HMGB1, further studies are required. For example, the use of different isoforms of recombinant HMGB1 or knock down analysis of HMGB1 receptors are needed in this research field. In addition, mostly, *in vivo* and *in vitro* studies have been included in this review; thus, further clinical studies, such as a translational study using anti-HMGB1 antibody or HMGB1 protein as a therapeutic agent, are needed to examine the biological effects of HMGB1 in the human body.

## Author Contributions

KY contributed to the conception, design, analysis, and interpretation of the study and wrote the manuscript. HI and HA contributed to the analysis and interpretation of the study and drafted the manuscript. HA, CY-H, AH, RS-K, and YZ contributed to *in vivo* experiments. HW and TY contributed to the interpretation of the study. MN and ST contributed to the conception, design, analysis, and interpretation of the study and drafted and critically revised the manuscript. All authors contributed to the article and approved the submitted version.

## Conflict of Interest

The authors declare that the research was conducted in the absence of any commercial or financial relationships that could be construed as a potential conflict of interest.
